# Prevalence of Anemia and Its Associated Risk Factors Among 6-Months-Old Infants in Beijing

**DOI:** 10.3389/fped.2019.00286

**Published:** 2019-07-12

**Authors:** Qinrui Li, Furong Liang, Weilan Liang, Wanjun Shi, Ying Han

**Affiliations:** Department of Pediatrics, Peking University First Hospital, Beijing, China

**Keywords:** iron deficiency anemia, growth and development, infants, Denver Development Screen Test (DDST), feeding style

## Abstract

**Objective:** The worldwide prevalence of anemia is ~24.8%. Iron deficiency anemia is common in children and women and associated with sensory, motor, cognitive, language, and socioemotional deficits. Therefore, detection and early intervention strategies for anemia in infants are urgently needed. To prevent the occurrence of iron deficiency anemia, we aimed to identify risk factors associated with anemia in infants.

**Methods:** This investigation involved a cross-sectional study of 6-months-old infants discharged between April 2014 and September 2017 from Peking University First Hospital. We assessed birth information, maternal age, and maternal educational level as well as data on feeding style, complementary foods and primary caregivers. The infants were assessed with the Denver Developmental Screening Test (DDST).

**Results:** A total of 1,127 6-months-old infants were enrolled at the hospital. We found that the prevalence of anemia among infants in Beijing was ~11.8%. Premature infants had a higher rate of anemia than full-term infants (χ^2^ = 40.103, *P* < 0.001). Infants born in autumn or winter were at an elevated risk of developing anemia (χ^2^ = 22.949, *P* < 0.001). Birth weight had no effect on the rate of anemia in infants (χ^2^ = 0.023, *P* = 0.568). Infants who were exclusively breastfeeding had higher anemia rates than those who were fed formula (χ^2^ = 38.466, *P* < 0.001). Infants whose caregivers added no complementary foods had higher anemia rates (24.7%) than those whose caregivers added more than two kinds of complementary food (8.2%). The type of caregiver had no effect on the anemia rate in infants (χ^2^ = 0.031, *P* = 1.000).

**Conclusions:** The following factors resulted in a higher prevalence of anemia in our study a gestational age at birth of <37 weeks, exclusive breastfeeding, a lack of supplementation with complementary foods and a spring birth date. No significant differences in DDST pass rates were evident between infants with and without anemia.

## Background

Anemia is a common disease that affects ~1.6 billion people worldwide, especially infants and women. The World Health Organization (WHO) has estimated that the global prevalence of anemia to be ~24.8% (1). Anemia is defined as a hemoglobin (Hb) concentration that is two standard deviations below the mean for the patient's age. The factors associated with anemia may include genetics, chronic infections, and nutritional deficiencies, such as hemoglobinopathies, iron deficiency, folate deficiency, and vitamin B12 deficiency. Iron deficiency anemia is common in children, and iron deficiency has a very important influence on infant neurological development. Iron is an essential factor in neuronal myelination, metabolism, neurotransmission and neurogenesis, and it affects behavior, memory, learning and sensory systems (2, 3). In rodents, iron deficiency alters the metabolome in the striatum and delays behavioral development (4). Iron deficiency also alters the neurochemical profile associated with cognitive function in the developing hippocampus (5). Iron deficiency in infancy is associated with impaired mental and motor development, especially in language capabilities, bodily balance and ordination skills (6, 7). Morath and Mayer-Proschel (8) found that iron deficiency during pregnancy affected the function of glial precursor cells in rats. Iron is essential for multiple enzymes associated with the synthesis of neurotransmitters, including dopamine and norepinephrine, which are associated with learning and memory function (9). Iron is important for multiple electron transfer reactions associated with brain energy metabolism (10). Perinatal iron deficiency reduces neuronal activity, especially in the hippocampal region, which is associated with memory function (11).

Infants aged 6–12 months are at an elevated risk of anemia because they are developing and growing rapidly and because the stored iron from the mother may be deficient. The addition of complementary food during this period is important. Complementary foods influence the overall nutritional status of the infant. The risk of iron deficiency increases in later infancy if infants are exclusively breastfeeding (12). A study of infants in poor rural areas of China showed that complementary food supplements could reduce the prevalence of anemia (13). Moreover, home food fortification with iron increased Hb levels and decreased anemia rates (14). Hong et al. (15) showed that the combination of prolonged breastfeeding and an inadequate supply of red meat results in iron deficiency and iron deficiency anemia. Insufficient complementary feeding behavior is associated with undernutrition, which results in poor growth and cognitive development. Baye et al. (16) found that positive, responsive maternal feeding behavior was positively associated with Hb concentrations. Other factors also influence the iron status of infants; one example is the maternal iron status, which is associated with anemia in children. In this case, anemia occurs because an infant cannot obtain enough iron from the stored iron transferred from the mother or from breast milk (17). The educational level of the mother or caregiver is also associated with the anemia rate (18, 19).

Rapid economic development and the acceleration of industrialization in China have led to major changes in Chinese lifestyles, especially for new parents. As the capital city of China, Beijing is more representative of such changes than other cities. Infants in Beijing consume increasingly rich diets, and their developmental health is better now than in the past. As the education level of the mother increases, her knowledge of how to feed her children improves. In the present study, we aimed to investigate the factors currently associated with infant anemia in Beijing, with the goal of improving the health of these infants.

## Methods

### Participants

This investigation involved a cross-sectional study conducted at Peking University First Hospital. The participants were enrolled between April 2014 and September 2017. The infants included in this study were 6 months old and did not have severe disease or any abnormality at birth. The exclusion criteria were as follows: younger or older than 6 months old, a history of asphyxia at birth, and a history of severe disease. Children who met the inclusion criteria and did not meet the exclusion criteria were enrolled in our study. For each infant, we collected data regarding sex (male or female), maternal education level (less than undergraduate, undergraduate, or more than undergraduate), birth weight, birth season, caregivers (parent, grandparent, or babysitter), feeding style (exclusive breastfeeding, mixed feeding or formula feeding), and complementary food usage (none, one kind of complementary food or two or more kinds of complementary food). We defined the four seasons as follows: winter (December, January, and February), spring (March, April, and May), summer (June, July, and August), and autumn (September, October, and November).

### Diagnostic Criteria and Classification

Anemia is defined as an Hb concentration <110 g/L according to the WHO diagnostic criteria. Mild anemia is defined as an Hb concentration between 90 and 110 g/L, moderate anemia is defined as an Hb concentration between 60 and 90 g/L, and severe anemia is defined as an Hb concentration <60 g/L. Iron deficiency anemia is defined as a mean cell volume (MCV) <80 fl, mean cell hemoglobin (MCH) <27 pg, and mean corpuscular Hb concentration (MCHC) <310 g/L.

### Assessment of Ability Development

The development of the infants' intelligence was assessed with the Denver Developmental Screening Test (DDST). The DDST was standardized for Chinese use in 1982 and has been utilized worldwide to assess the intelligence development of children aged 1 month to 6 years. The standardized DDST consists of 104 items and covers four areas of development: (a) personal/social, (b) fine motor/adaptive, (c) language, and (d) gross motor. In the present study, three trained professionals examined the children. The response options for the items were “passes,” “fails,” “refuses,” and “has not had the opportunity.” The results of the DDST could be normal (no delays), suspect (2 or more caution items and/or 1 or more delays), abnormal (2 or more delays) or untestable (refusal of one or more items completely to the left of the age line or more than one item intersected by the age line in the 75–90% area). The children with suspect or abnormal results were retested 2 or 3 weeks later.

### Statistical Analysis

The data were analyzed with SPSS 18.0. Numerical variables are presented as the mean ± standard deviation (SD) (birth weight). Enumeration data and ranked data are presented as percentages. ANOVA, the χ^2^ test and non-parametric tests were used to assess the differences in child development between the three groups. A *P*-value < 0.05 was considered statistically significant.

### Ethics

The study was carried out in accordance with recommendations of the Clinical Research Ethics Committee of Peking University First Hospital (Permit Number: 2017 [1375]). All parents provided written informed consent before the start of the study.

## Results

A total of 1,127 infants (591 male and 536 female) aged 6 months were included in this study. The average birth weights of the infants were 3.38 ± 0.42 kg for males and 3.26 ± 0.45 kg for females. The average birth lengths were 50.6 ± 2.1 cm for males and 50.2 ± 1.8 cm for females. The average Hb levels were 117.9 ± 8.5 g/L in males and 118.4 ± 7.8 g/L in females (Table 1). The mean maternal age was ~31.8 ± 3.5 years.

**Table 1:** Infant birth information.

**Sex**	***N***	**Birth weight (kg)**	**Birth length (cm)**	**Hemoglobin (g/L)**
Male	591	3.38 ± 0.42	50.6 ± 2.1	117.9 ± 8.5
Female	536	3.26 ± 0.45	50.2 ± 1.8	118.4 ± 7.8

Table 2 contains the demographic information (e.g., maternal educational level, maternal age, gestational age at birth, and birth season) and Hb levels of the included infants. The mean Hb value was 118.2 ± 8.1 g/L (range 80.0–146.0 g/L). A total of 133 (11.8%) infants had microcytic hypochromic anemia (MCV <80 fl, MCH <27 pg, and MCHC <310 g/L), including 126 (11.2%) with mild anemia (104.6 ± 4.7 g/L) and 7 (0.6%) with moderate anemia (85.3 ± 3.1 g/L). No infants displayed severe anemia. The mean Hb level in the non-anemia group was 120.1 ± 6.1 g/L. The mean Hb values of the groups with maternal educational levels of less than undergraduate, undergraduate and more than undergraduate were 118.0 ± 8.9, 118.7 ± 7.5, and 117.4 ± 8.5 g/L, respectively. The ages of the mothers ranged from 22 to 45 years old. The effects of maternal age on the Hb levels of the infants are shown in Table 2. The study group contained 65 premature infants, whose mean Hb level was 113.3 ± 10.3 g/L. The study group contained 1,062 full-term infants, whose mean Hb level was 118.5 ± 7.9 g/L. Table 2 also shows the effects of birth season and birth weight on Hb levels.

**Table 2:** Demographic information and hemoglobin levels.

**Characteristics**	***n***	**Percent (%)**	**Hemoglobin (g/L)**
**Hemoglobin (g/L)**			
Normal (>110)	994	88.2%	120.1 ± 6.1
Mild (90–109)	126	11.2%	104.6 ± 4.7
Moderate (60–89)	7	0.6%	85.3 ± 3.1
Severe (<59)	0	0	–
**Maternal educational level**			
Less than undergraduate	185	17.6%	118.0 ± 8.9
Undergraduate	580	55.2%	118.7 ± 7.5
More than undergraduate	285	27.1%	117.4 ± 8.5
**Maternal age**			
<25 years	8	0.7%	114.9 ± 6.6
25–29 years	289	26.9%	118.3 ± 8.1
30–34 years	556	51.8%	118.6 ± 7.8
35–39 years	187	17.4%	117 5 ± 8.4
>39 years	33	3.1%	116.8 ± 8.7
**Gestational age at birth**			
<37 weeks	65	5.8%	113.3 ± 10.3
>37 weeks	1,062	94.2%	118.5 ± 7.9
**Birth season**			
Spring	220	19.5%	117.1 ± 7.4
Summer	292	25.9%	118.1 ± 5.3
Autumn	336	29.8%	118.3 ± 9.5
Winter	279	24.8%	119.0 ± 9.2
**Birth weight**			
<2,500 g	36	3.3%	118.5 ± 9.5
>2,500 g	1,063	96.7%	118.1 ± 8.1

As shown in Table 3, feeding practices affected the infants' Hb levels. A total of 197 (17.5%) infants were fed formula and had a mean Hb level of 120.7 ± 7.3 g/L. A total of 634 (56.3%) infants were exclusively fed breast milk and had a mean Hb level of 116.6 ± 8.5 g/L. A total of 296 (26.3%) infants received mixed feeding and had a mean Hb level of 119.9 ± 7.0 g/L. Most infants (96.3%) had diets containing complementary foods as follows: one type of complementary food (rice flour) or two or more types of complementary foods (rice flour, yolk or liver paste). A total of 202 (41.6%) infants, 253 (52.1%) infants, and 31 (6.4%) infants were cared for by their parents, grandparents and babysitters, respectively, and the mean Hb levels of these infants were 117.3 ± 7.6, 117.7 ± 8.2, and 118.3 ± 9.7 g/L, respectively.

**Table 3:** Infant feeding practices.

**Feeding practice**	***n***	**Percent (%)**	**Hemoglobin (g/L)**
**Feeding style**			
Exclusive breastfeeding	634	56.3%	116.6 ± 8.5
Mixed feeding	296	26.3%	119.9 ± 7.0
Artificial feeding	197	17.5%	120.7 ± 7.3
**Complementary foods**			
None	85	3.7%	115.7 ± 11.7
One kind	390	32.3%	119.0 ± 8.9
More than two kinds	478	64.0%	119.1 ± 7.0
**Caregivers**			
Parents	202	41.6%	117.3 ± 7.6
Grandparents	253	52.1%	117.7 ± 8.2
Babysitters	31	6.4%	118.3 ± 9.7

The factors that affected infant anemia are shown in Table 4. Gestational age at birth, birth season, feeding style and complementary food supplementation had clear effects on infant anemia. Premature infants had higher rates of anemia than full-term infants (χ^2^ = 40.103, *P* < 0.001). The infants born in autumn or winter were at an increased risk of developing anemia (χ^2^ = 22.949, *P* < 0.001). Birth weight had no effect on the rate of anemia in infants (χ^2^ = 0.023, *P* = 0.568). Infants who were exclusively breastfeeding had higher anemia rates than infants who were fed formula (χ^2^ = 38.466, *P* < 0.001). Infants whose caregivers added no complementary foods had higher anemia rates (24.7%) than infants whose caregivers added two or more types of complementary food (8.2%). The type of caregiver had no effect on infant anemia rates (χ^2^ = 0.031, *P* = 1.000). Table 5 shows the multivariate logistic regression analysis results of the risk factors for infant anemia. Gestational age at birth, birth season, feeding style and complementary food supplementation significantly affected infant anemia rates (*P* < 0.05).

**Table 4:** Factors associated with infant anemia.

**Factors**	**Anemia *N* (%)**	**Non-anemia *N* (%)**	**χ^**2**^**	***P***
			1.799	0.196
Male	77 (13%)	514 (87%)		
Female	56 (10.4%)	480 (89.6%)		
**Maternal educational level**			2.132	0.352
Less than undergraduate	24 (13%)	161 (87%)		
Undergraduate	61 (10.5%)	519 (89.5%)		
More than undergraduate	39 (13.7%)	246 (86.3%)		
**Maternal age**			3.147	0.672^a^
<25 years	1 (12.5%)	7 (87.5%)		
25–29 years	38 (13.1%)	251 (86.9%)		
30–34 years	58 (10.4%)	498 (89.6%)		
35–39 years	22 (11.8%)	165 (88.2%)		
40–44 years	5 (15.6%)	27 (84.4%)		
>44 years	0 (0%)	1 (100%)		
**Gestational age at birth**			40.103	0.000^*^
<37 weeks	25 (38.5%)	40 (61.5%)		
>37 weeks	108 (10.2%)	954 (89.8%)		
**Birth season**			22.949	0.000^*^
Spring	13 (5.9%)	207 (94.1%)		
Summer	22 (7.5%)	270 (92.5%)		
Autumn	51 (15.2%)	285 (84.8%)		
Winter	47 (16.8%)	232 (83.2%)		
**Birth weight**			0.023	0.568
<2,500 g	4 (11.1%)	32 (88.9%)		
>2,500 g	127 (11.9%)	936 (88.1%)		
**Feeding style**			38.466	0.000^*^
Exclusive breastfeeding	108 (17%)	526 (83%)		
Mixed feeding	13 (4.4%)	283 (95.6%)		
Artificial feeding	12 (6.1%)	185 (93.9%)		
**Complementary foods**			21.509	0.000^*^
None	21 (24.7%)	64 (75.3%)		
One kind	51 (13.1%)	339 (86.9%)		
More than two kinds	44 (8.2%)	495 (91.8%)		
**Caregivers**			0.031	1.000
Parents	27 (13.4%)	175 (86.6%)		
Grandparents	34 (13.4%)	219 (86.6%)		
Babysitters	4 (12.9%)	27 (87.1%)		

a *Fisher's exact test*.

* *P < 0.001*.

**Table 5:** Univariate analysis of factors influencing infant anemia.

**Factors**	***B***	**SE**	***P***	**OR**	**95% CI for Exp (B)**
Gestational age at birth	−1.979	0.303	0.000^*^	0.138	0.076–0.250
Birth season	−0.473	0.098	0.000^*^	0.623	0.515–0.755
Feeding style	0.847	0.169	0.000^*^	2.332	1.675–3.247
Complementary foods	0.254	0.096	0.008^*^	1.289	1.068–1.554

*B, coefficient; SE, standard error; OR, odds ratio, which equals to the power of the coefficient B; 95% CI for Exp (B), 95% confidence interval of the exponentiation of the coefficient B*.

* *P < 0.001*.

As shown in Table 6, anemia had no significant effect on the DDST pass rates (χ^2^ = 5.600, *P* = 0.051).

**Table 6:** Effect of anemia on DDST pass rates.

	**Pass**	**Suspect**	**Abnormal**	**χ^**2**^**	***P***
Anemia	121 (91.0%)	8 (6.0%)	4 (3.0%)	5.600	0.051
Non-anemia	910 (91.5%)	77 (7.7%)	7 (0.7%)		

## Discussion

The present study examined information associated with 1,127 6-months-old infants and revealed an anemia prevalence of 11.8%. In our study, the risk factors associated with anemia were gestational age at birth, birth season, feeding style and complementary food supplementation. No significant difference in the DDST pass rate was evident between infants with and without anemia.

The anemia rate in our study was lower than the global anemia rate (24.8%) (1). The WHO has estimated that anemia affects 1.62 billion people globally, including 293 million preschool-aged children, 56 million pregnant women and 468 million non-pregnant women. A total of 54.3% of infants aged 6–11 months are reportedly anemic, and 24.3% of infants in rural China suffer from moderate or severe anemia (20). Although the prevalence of anemia in China has gradually decreased, the adverse impacts of anemia on infants and society are profound. The most common form of anemia is iron deficiency anemia (21). Iron deficiency in children <3 years of age negatively affects their physical and intellectual development (22). Additionally, the prevalence of anemia is highest at the ages of 6–12 months, a period that is critical for psychomotor development. Some studies have shown that infants with iron deficiency have lower auditory brainstem response (ABR) responses than those with normal iron levels, representing the iron deficiency anemia infants with delayed central nervous system (CNS) myelination (23). Therefore, we aimed to identify the risk factors associated with anemia.

In our study, we found that premature infants had an increased risk of developing anemia at 6 months of age, which was consistent with other studies (24, 25). Halliday et al. (26) found that 26% of premature infants had iron deficiency during the first year of life. Preterm infants are at high risk of nutritional deficiency because they have low stores of iron, zinc and vitamin A (27). Preterm infants can experience blood loss at birth, inadequate erythropoiesis, blood sampling, rapid growth, hemorrhage and hemolysis. Therefore, most premature infants have smaller blood volumes and experience more profound anemia than full-term infants (28). The effects of iron deficiency include poor physical growth, gastrointestinal disturbances, neurodevelopmental impairments and altered immunity (29, 30). Therefore, premature infants should receive iron supplementation from sources, such as fortified human milk, iron-fortified formula or medicinal elemental iron (e.g., 2–4 mg/kg/d).

We showed that infants born in spring had lower Hb levels than infants born in winter. A previous study also showed that the incidence of anemia in infants aged 5–7 months who were born in spring and summer was higher than that in infants aged 5–7 months who were born in autumn or winter (31). This difference is probably due to seasonal variations in the folate and vitamin B6 statuses among women who may be attempting to become pregnant (32, 33).

In our study, we found that feeding style and complementary foods affected the prevalence of anemia in infants. Infants who were exclusively breastfeeding (17%) had a higher prevalence of anemia than infants whose diets were mixed or infants who were fed formula (4.4 and 6.1%). The formula consumed by the infants contained iron; therefore, the infants whose diets were mixed and the infants who were fed formula were unlikely to develop anemia. The addition of two or more types of complementary foods was associated with the lowest prevalence of anemia among the three groups (8.2 vs. 24.7% and 13.1%). The WHO recommends exclusive breastfeeding without the introduction of any nutritious complementary foods for the first 6 months (34). The concentration of iron in human milk is relatively low. In China, some parents do not add complementary foods or iron supplementation during the first 6 months, which has become an important cause of infant anemia. As the infant grows, iron from human milk becomes insufficient to meet the increasing needs of the body tissue and circulation (35). Therefore, complementary foods containing iron should be given to infants at the proper time to avoid anemia (36). In our study, the infants who were fed one type of food were always fed rice flour, and the infants who were fed two or more types of complementary foods were always fed liver paste, yolk or meat paste, which contain high levels of iron. Wang et al. (37) found that the introduction of complementary foods comprising rice cereal, porridge, and bread was more likely to result in the development of anemia than the introduction of animal-based foods. Rice cereal, porridge, and bread contain low amounts of bioavailable iron and have phytates that inhibit iron absorption (38). One study showed that deficiencies in vitamin A, vitamin C, zinc and iron were associated with the late introduction of complementary food (39). Thus, the addition of complementary foods should begin when maternal milk no longer meets the nutritional needs of the infant. Complementary foods not only provide nutrition to infants but also shape their future eating habits (40). However, some studies have shown that the early introduction of complementary foods is associated with allergies (41). Conversely, the late introduction of complementary foods is associated with developmental delays, such as motor skill deficits (42). In subsequent studies, we intend to identify a suitable time for the introduction of complementary foods.

In our study, we found that anemia was not associated with the DDST pass rates. This lack of association was probably because the duration of anemia in the infants was not sufficient to influence the DDST results. Iron requirements are most likely to exceed iron intake during the first 6–18 months of life because infants' growth and blood volume expansion proceed rapidly during this time (43). In our study, we investigated 6-months-old infants with anemia; thus, anemia had not been present for long. Lozoff et al. (44) showed that infants with iron deficiency anemia processed information slower at 12 months of age than infants with a good iron status. Infants with iron deficiency anemia have reduced dopamine function at 9 months, and this condition worsens at 12 months (45). Shafir et al. (46) found that 12- to 23-months-old infants with iron deficiency anemia did not catch up in motor development, although iron therapy during infancy corrected their anemia. In summary, anemia in infants should be detected as soon as possible. We examined Hb levels at the age of 6 months to select anemic infants and provide therapy early, thus preventing unfavorable outcomes caused by iron deficiency. Some studies have shown that at 6–8 weeks after birth, infants should receive iron supplementation (2–3 mg/kg per day) or formula containing iron (12 mg/L) to prevent iron deficiency anemia. Infants with birth weights below 1,000 g require additional iron (47).

Our study showed that the gestational age at birth, birth season, feeding style and complementary food supplementation affected anemia in infants aged 6 months. Early detection is of utmost importance to prevent adverse outcomes caused by infant anemia. Next, the caregiver should add iron-containing complementary foods, such as liver paste, yolk and meat paste, at a suitable time, especially in infants whose gestational age at birth is <37 weeks. In addition, infants born during different seasons should be supplied with nutrition accordingly. For example, infants born during spring should be provided with more iron than those born during winter.

Nevertheless, our study had several limitations. First, China is an expansive country that contains individuals of different ethnicities who inhabit different geographic locations and have various dietary traditions. These factors are probably associated with the prevalence of anemia. However, our study was limited to the population in Beijing. Further studies should focus on low-income and middle-income provinces in China. Second, we also lacked information regarding maternal anemia. Maternal anemia has been associated with infant anemia (48). In future studies, we will examine data on maternal anemia.

## Conclusions

Anemia is a global public health problem that influences infant development, resulting in poor outcomes in adulthood. The risk factors identified in our study, such as a gestational age at birth of <37 weeks, exclusive breastfeeding, a lack of supplementation with complementary foods and a spring birth date, may be meaningful for the early detection of infant anemia and the prompt delivery of interventions.

## Data Availability

The raw data supporting the conclusions of this manuscript will be made available by the authors, without undue reservation, to any qualified researcher.

## Ethics Statement

The study was carried out in accordance with the recommendations of the Clinical Research Ethics Committee of Peking University First Hospital. All subjects gave written informed consent in accordance with the Declaration of Helsinki. The protocol was approved by the Ethics Committee of Peking University First Hospital, China.

## Author Contributions

QL conducted the experiments, analyzed the data, wrote the manuscript, and approved the final version to be published. YH contributed to the conception and design of the experiment, acquired the data, revised the manuscript, approved the final version to be published, and agreed to be accountable for all aspects of the work. FL and WL contributed to the conception and design of the experiment, acquired the data, critically revised the manuscript, and approved the final version to be published. WS contributed to the conception and design of the experiment and approved the final version to be published.

### Conflict of Interest Statement

The authors declare that the research was conducted in the absence of any commercial or financial relationships that could be construed as a potential conflict of interest.

## References

[1] McLean E Cogswell M Egli I Wojdyla D de Benoist B. Worldwide prevalence of anaemia, WHO Vitamin and mineral nutrition information system, 1993–2005. Public Health Nutr. (2009) 12:444–54. 10.1017/S1368980008002401 18498676

[2] Ortiz E Pasquini JM Thompson K Felt B Butkus G Beard J. Effect of manipulation of iron storage, transport, or availability on myelin composition and brain iron content in three different animal models. J Neurosci Res. (2004) 77:681–9. 10.1002/jnr.20207 15352214

[3] Lozoff B. Iron deficiency and child development. Food Nutr Bull. (2007) 28:S560–71. 10.1177/15648265070284S409 18297894

[4] Ward KL Tkac I Jing Y Felt B Beard J Connor J. Gestational and lactational iron deficiency alters the developing striatal metabolome and associated behaviors in young rats. J Nutr. (2007) 137:1043–9. 10.1093/jn/137.4.1043 17374674 PMC1892181

[5] Rao R Tkac I Townsend EL Gruetter R Georgieff MK. Perinatal iron deficiency alters the neurochemical profile of the developing rat hippocampus. J Nutr. (2003) 133:3215–21. 10.1093/jn/133.10.3215 14519813

[6] Walter T Kovalskys J Stekel A. Effect of mild iron deficiency on infant mental development scores. J Pediatr. (1983) 102:519–22. 6834185 10.1016/s0022-3476(83)80177-2

[7] Lozoff B Brittenham GM Wolf AW McClish DK Kuhnert PM Jimenez E. Iron deficiency anemia and iron therapy effects on infant developmental test performance. Pediatrics. (1987) 79:981–95. 2438638

[8] Morath DJ Mayer-Proschel M. Iron deficiency during embryogenesis and consequences for oligodendrocyte generation *in vivo*. Dev Neurosci. (2002) 24:197–207. 10.1159/000065688 12401959

[9] Lozoff B Beard J Connor J Barbara F Georgieff M Schallert T. Long-lasting neural and behavioral effects of iron deficiency in infancy. Nutr Rev. (2006) 64:S34–43; discussion S72–91. 10.1301/nr.2006.may.s34-s43 16770951 PMC1540447

[10] Beard J. Iron deficiency alters brain development and functioning. J Nutr. (2003) 133:1468S−72S. 10.1093/jn/133.5.1468S 12730445

[11] de Deungria M Rao R Wobken JD Luciana M Nelson CA Georgieff MK. Perinatal iron deficiency decreases cytochrome c oxidase (CytOx) activity in selected regions of neonatal rat brain. Pediatric Res. (2000) 48:169–76. 10.1203/00006450-200008000-00009 10926291

[12] Clark KM Li M Zhu B Liang F Shao J Zhang Y. Breastfeeding, mixed, or formula feeding at 9 months of age and the prevalence of iron deficiency and iron deficiency anemia in two cohorts of infants in China. J Pediatric. (2017) 181:56–61. 10.1016/j.jpeds.2016.10.041 27836288 PMC5274569

[13] Zhang Y Wu Q Wang W van Velthoven MH Chang S Han H. Effectiveness of complementary food supplements and dietary counselling on anaemia and stunting in children aged 6-23 months in poor areas of Qinghai Province, China: a controlled interventional study. BMJ Open. (2016) 6:e011234. 10.1136/bmjopen-2016-011234 27799239 PMC5093399

[14] Huo J Sun J Fang Z Chang S Zhao L Fu P. Effect of home-based complementary food fortification on prevalence of anemia among infants and young children aged 6 to 23 months in poor rural regions of China. Food Nutr Bull. (2015) 36:405–14. 10.1177/0379572115616001 26612420

[15] Hong J Chang JY Shin S Oh S. Breastfeeding and red meat intake are associated with iron status in healthy Korean weaning-age infants. J Korean Med Sci. (2017) 32:974–84. 10.3346/jkms.2017.32.6.974 28480656 PMC5426231

[16] Baye K Tariku A Mouquet-Rivier C. Caregiver-infant's feeding behaviours are associated with energy intake of 9–11 month-old infants in rural Ethiopia. Matern Child Nutr. (2018) 14:e12487. 10.1111/mcn.12487 28675690 PMC6866062

[17] Zhao A Zhang Y Peng Y Li J Yang T Liu Z. Prevalence of anemia and its risk factors among children 6–36 months old in Burma. Am J Trop Med Hyg. (2012) 87:306–11. 10.4269/ajtmh.2012.11-0660 22855763 PMC3414569

[18] Abubakar A Uriyo J Msuya SE Swai M Stray-Pedersen B. Prevalence and risk factors for poor nutritional status among children in the Kilimanjaro region of Tanzania. Int J Environ Res Public Health. (2012) 9:3506–18. 10.3390/ijerph9103506 23202759 PMC3509468

[19] Ayoya MA Ngnie-Teta I Seraphin MN Mamadoultaibou A Boldon E Saint-Fleur JE. Prevalence and risk factors of anemia among children 6–59 months old in Haiti. Anemia. (2013) 2013:502968. 10.1155/2013/502968 23555053 PMC3608103

[20] Luo R Shi Y Zhou H Yue A Zhang L Sylvia S. Anemia and feeding practices among infants in rural Shaanxi Province in China. Nutrients. (2014) 6:5975–91. 10.3390/nu6125975 25533008 PMC4277010

[21] Martorell R Ascencio M Tacsan L Alfaro T Young MF Addo OY. Effectiveness evaluation of the food fortification program of Costa Rica: impact on anemia prevalence and hemoglobin concentrations in women and children. Am J Clin Nutr. (2015) 101:210–7. 10.3945/ajcn.114.097709 25527765 PMC5884061

[22] Black RE Victora CG Walker SP Bhutta ZA Christian P de Onis M. Maternal and child undernutrition and overweight in low-income and middle-income countries. Lancet. (2013) 382:427–51. 10.1016/S0140-6736(13)60937-X 23746772

[23] Amin SB Orlando M Eddins A MacDonald M Monczynski C Wang H. *In utero* iron status and auditory neural maturation in premature infants as evaluated by auditory brainstem response. J Pediatr. (2010) 156:377–81. 10.1016/j.jpeds.2009.09.049 19939407 PMC2827634

[24] Shaw JC. Iron absorption by the premature infant. The effect of transfusion and iron supplements on the serum ferritin levels. Acta Paediatr Scand Suppl. (1982) 299:83–9. 6963546 10.1111/j.1651-2227.1982.tb09630.x

[25] Rao R Georgieff MK. Iron therapy for preterm infants. Clin Perinatol. (2009) 36:27–42. 10.1016/j.clp.2008.09.013 19161863 PMC2657918

[26] Halliday HL Lappin TR McClure G. Iron status of the preterm infant during the first year of life. Biol Neonate. (1984) 45:228–35. 6722221 10.1159/000242009

[27] Shah MD Shah SR. Nutrient deficiencies in the premature infant. Pediatr Clin North Am. (2009) 56:1069–83. 10.1016/j.pcl.2009.08.001 19931064

[28] Jeon GW Sin JB. Risk factors of transfusion in anemia of very low birth weight infants. Yonsei Med J. (2013) 54:366–73. 10.3349/ymj.2013.54.2.366 23364969 PMC3575997

[29] Aggett PJ. Trace elements of the micropremie. Clin Perinatol. (2000) 27:119–29, vi. 10.1016/S0095-5108(05)70009-9 10690567

[30] Lozoff B Georgieff MK. Iron deficiency and brain development. Semin Pediatr Neurol. (2006) 13:158–65. 10.1016/j.spen.2006.08.004 17101454

[31] Yalcin SS Dut R Yurdakok K Ozmert E. Seasonal and gender differences in hemoglobin value in infants at 5–7 months of age. Turk J Pediatr. (2009) 51:572–7. 20196391

[32] Zhang J Cai WW Chen H. Perinatal mortality in Shanghai: 1986–1987. Int J Epidemiol. (1991) 20:958–63. 1800437 10.1093/ije/20.4.958

[33] Ronnenberg AG Goldman MB Aitken IW Xu X. Anemia and deficiencies of folate and vitamin B-6 are common and vary with season in Chinese women of childbearing age. J Nutr. (2000) 130:2703–10. 10.1093/jn/130.11.2703 11053510

[34] Ye F Chen ZH Chen J Liu F Zhang Y Fan QY. Chi-squared automatic interaction detection decision tree analysis of risk factors for infant anemia in Beijing, China. Chin Med J (Engl). (2016) 129:1193–9. 10.4103/0366-6999.181955 27174328 PMC4878165

[35] Tsai SF Chen SJ Yen HJ Hung GY Tsao PC Jeng MJ. Iron deficiency anemia in predominantly breastfed young children. Pediatr Neonatol. (2014) 55:466–9. 10.1016/j.pedneo.2014.02.005 24953965

[36] Krebs NF Hambidge KM. Complementary feeding: clinically relevant factors affecting timing and composition. Am J Clin Nutr. (2007) 85:639S−45S. 10.1093/ajcn/85.2.639S 17284770

[37] Wang F Liu H Wan Y Li J Chen Y Zheng J. Age of complementary foods introduction and risk of anemia in children aged 4–6 years: a prospective birth cohort in China. Sci Rep. (2017) 7:44726. 10.1038/srep44726 28333130 PMC5363060

[38] Zimmermann MB Chaouki N Hurrell RF. Iron deficiency due to consumption of a habitual diet low in bioavailable iron: a longitudinal cohort study in Moroccan children. Am J Clin Nutr. (2005) 81:115–21. 10.1093/ajcn/81.1.115 15640469

[39] Wutich A McCarty C. Social networks and infant feeding in Oaxaca, Mexico. Matern Child Nutr. (2008) 4:121–35. 10.1111/j.1740-8709.2007.00122.x 18336645 PMC6860882

[40] Pantoja-Mendoza IY Melendez G Guevara-Cruz M Serralde-Zuniga AE. Review of complementary feeding practices in Mexican children. Nutr Hosp. (2014) 31:552–8. 10.3305/nh.2015.31.2.7668 25617535

[41] Grimshaw KE Maskell J Oliver EM Morris RC Foote KD Mills EN. Introduction of complementary foods and the relationship to food allergy. Pediatrics. (2013) 132:e1529–38. 10.1542/peds.2012-3692 24249826

[42] Lutter CK. Macrolevel approaches to improve the availability of complementary foods. Food Nutr Bull. (2003) 24:83–103. 10.1177/156482650302400105 12664528

[43] Beard JL. Why iron deficiency is important in infant development. J Nutr. (2008) 138:2534–6. 10.1093/jn/138.12.2534 19022985 PMC3415871

[44] Lozoff B De Andraca I Castillo M Smith JB Walter T Pino P. Behavioral and developmental effects of preventing iron deficiency anemia in healthy full-term infants. Pediatrics. (2003) 112:846–54. 14523176

[45] Lozoff B Armony-Sivan R Kaciroti N Jing Y Golub M Jacobson SW Eye-blinking rates are slower in infants with iron deficiency anemia than in nonanemic iron-deficient or iron-sufficient infants. J Nutr. (2010) 140:1057–61. 10.3945/jn.110.120964 20335633 PMC2855268

[46] Shafir T Angulo-Barroso R Calatroni A Jimenez E Lozoff B. Effects of iron deficiency in infancy on patterns of motor development over time. Hum Mov Sci. (2006) 25:821–38. 10.1016/j.humov.2006.06.006 17050023 PMC1993818

[47] Siimes MA. Iron requirement in low birthweight infants. Acta Paediatr Scand Suppl. (1982) 296:101–3. 6961731 10.1111/j.1651-2227.1982.tb09606.x

[48] Meinzen-Derr JK Guerrero ML Altaye M Ortega-Gallegos H Ruiz-Palacios GM Morrow AL. Risk of infant anemia is associated with exclusive breast-feeding and maternal anemia in a Mexican cohort. J Nutr. (2006) 136:452–8. 10.1093/jn/136.2.452 16424127

