# Corrigendum: Prevalence of Anemia and Its Associated Risk Factors Among 6-Months-Old Infants in Beijing

**DOI:** 10.3389/fped.2019.00416

**Published:** 2019-10-18

**Authors:** Qinrui Li, Furong Liang, Weilan Liang, Wanjun Shi, Ying Han

**Affiliations:** Department of Pediatrics, Peking University First Hospital, Beijing, China

**Keywords:** iron deficiency anemia, growth and development, infants, Denver Development Screen Test (DDST), feeding style

In the published article, there was an error in the affiliations. “Qinrui Li” should only have the affiliation “Department of Pediatrics, Peking University First Hospital, Beijing, China”. The affiliation list has, therefore, been updated accordingly.

The authors apologize for this error and state that this does not change the scientific conclusions of the article in any way. The original article has been updated.

